# Short- and Long-Term Outcomes of Totally Versus Hybrid Minimally Invasive Ivor Lewis Oesophagectomy for Oesophageal Cancer: A Propensity Score-Matched Analysis

**DOI:** 10.3389/fonc.2022.849250

**Published:** 2022-05-26

**Authors:** Yi-Min Gu, Han-Lu Zhang, Yu-Shang Yang, Yong Yuan, Yang Hu, Guo-Wei Che, Long-Qi Chen, Wen-Ping Wang

**Affiliations:** Department of Thoracic Surgery, West China Hospital of Sichuan University, Chengdu, China

**Keywords:** oesophageal cancer, Ivor Lewis, totally minimally invasive oesophagectomy, hybrid, outcomes

## Abstract

**Background:**

Few objective studies have compared totally minimally invasive Ivor Lewis oesophagectomy with hybrid procedure. Here we investigated whether the choice between totally and hybrid minimally invasive Ivor Lewis oesophagectomy influenced short-term outcomes and long-term patient survival.

**Methods:**

Patients who underwent totally or hybrid minimally invasive Ivor Lewis oesophagectomy between January 2014 and December 2017 were propensity score matched in a 1:1 ratio. The short- and long-term outcomes between the two groups were compared before and after matching.

**Results:**

Of 138 totally and 156 hybrid minimally invasive oesophagectomy patients were eligible, 104 patients from each group were propensity score matched. Totally minimally invasive oesophagectomy was associated significantly with less blood loss (median(IQR) 100(60–150) vs 120(120–200) ml respectively; P < 0.001), pneumonia (13.5 vs 25.0%; P = 0.035), pleural effusion (3.8 vs 13.5%; P = 0.014), and chest drainage (7.5(6–9) vs 8(7–9) days; P = 0.009) than hybrid procedure. There was no significant difference in 3-year overall survival rate and 3-year disease-free survival rate between the two group.

**Conclusions:**

Totally minimally invasive Ivor Lewis oesophagectomy may improve short-term outcomes and specifically reduce the incidence of pulmonary complications compared with hybrid procedure. The long-term overall survival and disease-free survival rates between the two groups were similar.

## Introduction

Oesophageal cancer is the seventh most common malignant tumour and the sixth leading cause of cancer-related deaths globally (1). The long-term survival for patients with oesophageal cancer remains poor despite therapeutic improvements (2). The primary treatment for patients with resectable oesophageal cancer remains surgical resection. Ivor Lewis oesophagectomy is a common surgical approach for patients with cancers in the middle or lower third of the oesophagus. Hybrid minimally invasive Ivor Lewis oesophagectomy (HILO) is widely used in clinical practice, not only for radical resection but also in terms of safety in anastomosis (3). With the application of enhanced recovery after surgery among patients undergoing oesophagectomy, totally minimally invasive Ivor Lewis oesophagectomy (TILO) has gained popularity in recent years because it greatly reduces trauma to patients (4).

TILO appears superior to open thoracotomy and laparotomy, with a significant reduction in postoperative complications (5). Similarly, the MIRO trial has shown that minimally invasive oesophagectomy might improve patients’ health-related quality of life mediated by reductions in postoperative complications compared with open oesophagectomy (6). Moreover, Gottlieb-Vedi et al. have found that the long-term survival after minimally invasive oesophagectomy compares well with open procedures and might even be better (7). However, considering the choice between TILO and HILO, no consensus has been reached, with only two retrospective studies with small sample sizes concluding that there was no difference in short-term outcomes between the two groups (8, 9). There is also limited evidence on long-term patient survival for these procedures.

The aim of this study is to compare short- and long-term outcomes of TILO versus HILO for patients with cancers in the middle or lower third of the oesophagus.

## Materials and Methods

### Study Design

We conducted a retrospective study comparing TILO with HILO in patients with carcinoma of the middle or lower third of the oesophagus who underwent Ivor Lewis oesophagectomy. The trial was performed following the ethical principles of the Declaration of Helsinki (10), and the manuscript complies with the STROBE statement for cohort studies (11). The study was approved by the Ethics Committee of West China Hospital of Sichuan University (No. 2019134). Informed Consent was waived.

### Patients

We screened consecutive patients who underwent Ivor Lewis oesophagectomy in our department between January 2014 and December 2017. The operations were performed by four independent surgical teams, and each surgeon had the experience of more than 200 minimally invasive oesophagectomies before this study. The use of neoadjuvant therapy was determined by multidisciplinary tumour board discussions.

Inclusion criteria included (1) oesophageal cancer in the middle or lower third of the oesophagus, or a junctional adenocarcinoma (Siewert’s type I and II); (2) a tumour and nodal status judged to be resectable; (3) no distant metastases (M0); and (4) upper abdominal and right thoracic approach (Ivor Lewis procedure).

Exclusion criteria included (1) other histologic types of tumour except for squamous cell carcinomas or adenocarcinomas; (2) coexistence of other malignancies; (3) comorbidity with other gastrooesophageal diseases; (4) any history of thoracic or abdominal surgery.

### Surgery

Ivor Lewis oesophagectomy consisted of an abdominal phase (mobilization of the stomach and abdominal lymph node dissection) and a thoracic phase (tumour resection, mediastinal lymph node dissection, and intrathoracic anastomosis). In the HILO group, laparoscopy was combined with thoracotomy. In the TILO group, laparoscopy combined with video-assisted or robot-assisted thoracoscopic oesophagectomy was used. The choice of anastomotic type was based on each surgeon’s preference and experience. The anastomosis was performed using circular or linear staplers or hand-sewn sutures. The anastomosis techniques were feasible and safe with low incidence of leakage, and details was available in our previous published literature (12, 13). The extent of lymphadenectomy included two-field lymph node dissections were conducted. For oesophagogastric junction tumour, superior mediastinal lymph node dissection was not conducted routinely.

### End Points

The following variables were collected to compare the two groups: basic characteristics, intraoperative index, postoperative major complications, mortality, and long-term outcomes. The postoperative complication after esophagectomy was defined according to Esophagectomy Complications Consensus Group system (14), of which the definition of pneumonia was based on temperature, leukocytosis, purulent secretions and radiologic evidence of infiltration. A major complication was referred to as grade II or higher Clavien-Dindo classification (15). Death within the first 30 days following surgery and in-hospital death were defined as operative mortality. We classified tumour stage according to the 7th edition of the TNM staging system of oesophageal cancer.

### Follow-up

We recorded tumour local recurrence, distant metastasis, and patient mortality and survival status. Overall survival was measured as the time from operation to death. Disease-free survival was calculated as the time to recurrence. Patients alive or lost to follow-up were censored at the date of last follow-up. The patients were followed up every 3 months for the first 2 years and every 6 months thereafter. Follow-up information was available until 3 years postoperatively or the date of death.

### Statistical Analysis

Normally distributed and non-normally distributed continuous variables were analyzed using Student’s t test or Mann–Whitney U test, respectively. Categorical variables were compared using Chi squared test or Fisher’s exact tests. Survival analysis was performed using the Kaplan–Meier method. Comparisons were adjusted with Bonferroni correction in subgroup analysis. A 1:1 propensity score-matching (PSM) approach was performed with a caliper width of 0.1 standard deviation using IBM SPSS Statistics (version 25; IBM Corp., Armonk, NY, USA) software with FUZZY (version 2.0.1) and PSM (version 2.0.1) extensions. Propensity scores were estimated using logistic regression based on age, sex, body mass index (BMI), forced expiratory volume in 1 s (FEV1), comorbidities, neoadjuvant therapy, histological type of tumour, clinical T stage, clinical N stage, and tumour location. Comparisons between matched groups were performed with McNemar test or paired t test. Statistical significance was set at a two-sided P value of <0.05.

## Results

**Figure 1:**
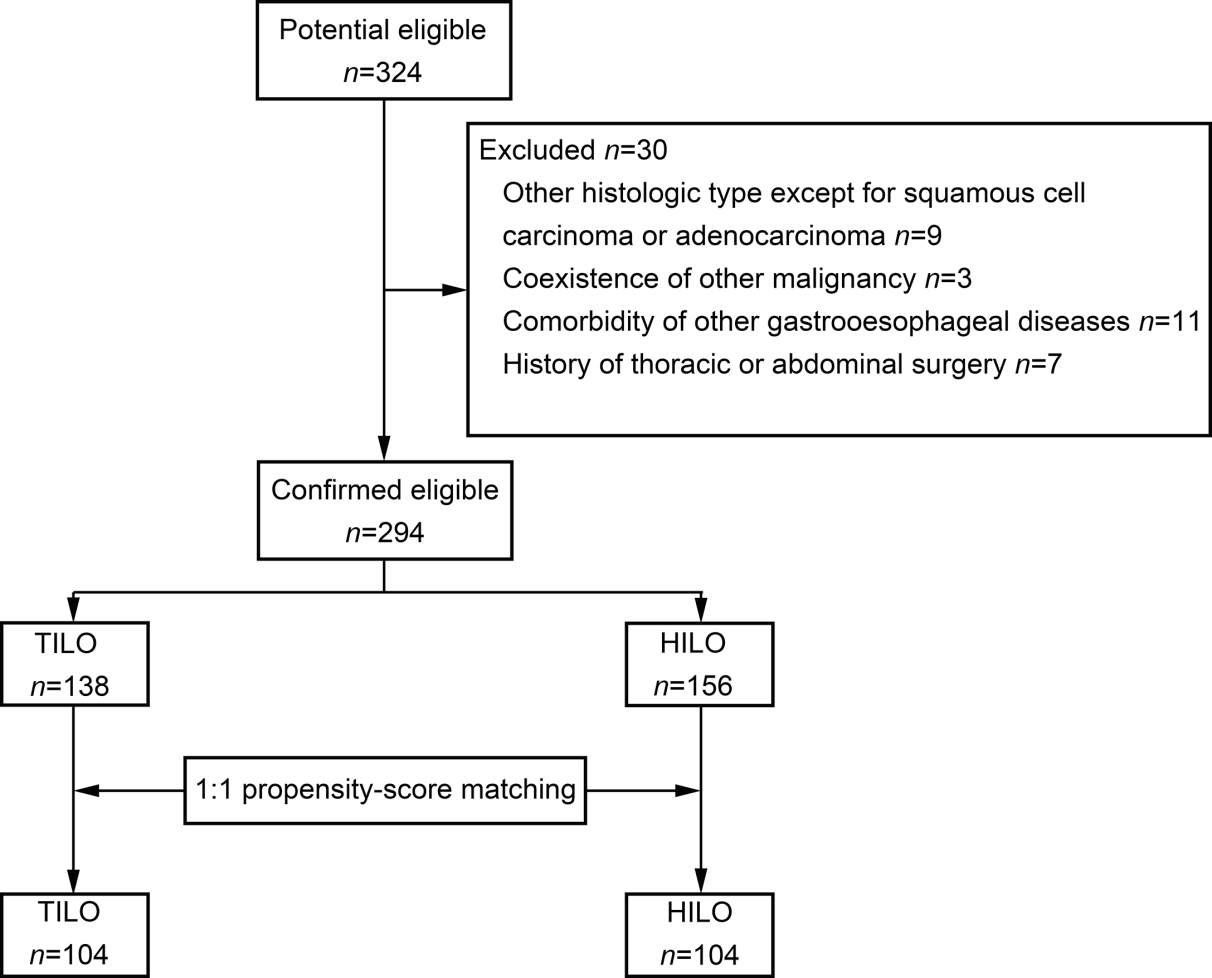
Flow diagram.

The patient selection flow diagram is shown in **Figure 1**. A total of 324 consecutive patients were screened. We excluded 30 patients (**Figure 1**). Finally, 294 patients were included in the analysis.

### Comparison of Short- and Long-Term Outcomes Between the Unmatched Groups

There were 138 patients who received TILO and 156 patients who received HILO in the unmatched analysis. The demographic and clinicopathological characteristics are shown in **Table 1**. Patients in TILO and HILO were comparable in age, sex, smoking history, BMI, FEV1, comorbidity and clinical N stage. However, the two groups differed significantly considering histological type of tumour (P = 0.011), clinical T stage (P = 0.009), and tumour location (P < 0.001).

**Table 1:** Baseline characteristics of patient population.

Characteristics	Unmatched dataset				1:1 Matched dataset			
	TILO	HILO	SD	*p* value	TILO	HILO	SD	*p* value
Sample size	138	156			104	104		
Age (years)	61.2 ± 7.9	62.9 ± 7.5	0.091	0.061	62.3 ± 7.6	61.6 ± 7.1	0.056	0.665
Sex ratio (M:F)	113:25	138:18	0.061	0.267	90:14	90:14	0.000	1.000
BMI (kg/m^2^)	22.8 ± 2.4	22.8 ± 2.5	0.001	0.902	22.9 ± 2.4	22.8 ± 2.4	0.001	0.576
History of smoking	67 (48.6)	76 (48.7)	0.097	0.994	54 (51.9)	46 (44.2)	0.019	0.267
FEV_1_ (%)	76.3 ± 17.2	74.6 ± 16.4	0.056	0.080	75.3 ± 17.9	76.0 ± 16.8	0.021	0.420
Comorbidities								
Hypertension	13 (9.4)	18 (11.5)	0.005	0.560	11 (10.6)	15 (14.4)	0.003	0.402
Diabetes mellitus	3 (2.2)	4 (2.5)	0.002	0.829	3 (2.9)	3 (2.9)	0.000	1.000
COPD	9 (6.5)	11 (7.1)	0.007	0.862	7 (6.7)	6 (5.8)	0.003	0.775
Neoadjuvant therapy	71 (51.4)	76 (48.7)	0.004	0.280	53 (50.9)	53 (50.9)	0.000	1.000
Histologic type				0.011				0.749
SCC	111 (80.4)	105 (67.3)	0.011		79 (76.0)	77 (74.0)	0.006	
Adenocarcinoma	27 (19.6)	51 (32.7)	0.121		25 (24.0)	27 (26.0)	0.008	
Clinical T stage				0.009				0.266
cT1	18 (13.0)	17 (10.9)	0.010		14 (13.5)	12 (11.5)	0.008	
cT2	29 (21.0)	14 (9.0)	0.091		19 (18.3)	13 (12.5)	0.020	
cT3	91 (66.0)	125 (80.1)	0.169		71 (68.2)	79 (76.0)	0.016	
Clinical N stage				0.128				0.958
cN0	61 (44.2)	52 (33.3)	0.011		47 (45.2)	45 (43.3)	0.008	
cN1	48 (34.8)	66 (42.3)	0.076		35 (33.7)	38 (36.5)	0.004	
cN2	29 (21.0)	38 (24.4)	0.046		22 (21.1)	21 (20.2)	0.001	
Tumour location				<0.001				0.780
Middle	39 (28.3)	54 (34.6)	0.062		34 (32.7)	34 (32.7)	0.001	
Lower	76 (55.1)	52 (33.3)	0.031		47 (45.2)	43 (41.3)	0.003	
Junctional	23 (17.4)	50 (32.1)	0.121		23 (22.1)	27 (26.0)	0.019	

Categoric data are shown as number (%) and continuous data as mean ± standard deviation; TILO, totally minimally invasive oesophagectomy; HILO, hybrid minimally invasive oesophagectomy; BMI, body mass index; FEV_1_, forced expiratory volume in 1 s; COPD, chronic obstructive pulmonary disease; SCC, squamous cell carcinoma; SD, standard difference.

In the unmatched dataset, we found that TILO was associated with less postoperative pneumonia (15.2 vs 25.0%; P = 0.038), and pleural effusion (4.3 vs 14.1%; P = 0.005) than the HILO group (**Table 2**). Furthermore, TILO was associated with shorter chest drainage than the HILO group (median(IQR) 8 (6–9) vs 8 (7–10.5) days; P = 0.003). The anastomotic leak rates were not significantly different between TILO and HILO (7.2% vs 6.4%, P = 0.772). No significant differences were observed in postoperative stay duration, anaemia requiring transfusion, mortality, being readmitted to the intensive care unit or unplanned reoperations between the two groups. One patient died from multiple organ failure within 30 days postoperatively in the TILO group, and one patients died from pulmonary infection within 90 days postoperatively in each group.

**Table 2:** Intraoperative and postoperative outcomes of patients with TILO versus HILO.

Characteristics	Unmatched dataset			1:1 Matched dataset		
	TILO	HILO	*p* value	TILO	HILO	*p* value
Sample size	138	156		104	104	
Operative time (min)	350 (350-350)	210 (180-210)	<0.001	350 (350-350)	210 (180-210)	<0.001
Estimated blood loss (mL)	100 (60-150)	120 (120-200)	<0.001	100 (60-150)	120 (120-200)	<0.001
No. of harvested lymph nodes	17 (12-23)	17 (12-22)	0.995	18 (12-23)	16.5 (11-23)	0.835
Upper mediastinal	1 (0-3)	0 (0-2)	0.020	1 (0-3)	0 (0-2)	0.066
Lower mediastinal	6 (4-9)	6 (4-9)	0.569	6 (4-9)	6 (4-10)	0.577
Abdominal	7 (4-15)	9 (5-13)	0.447	8 (5-15)	8.5 (5-13)	0.781
R0 resection	138 (100)	153 (100)	1.000	104 (100)	104 (100)	1.000
Major complications						
Pneumonia	21 (15.2)	39 (25.0)	0.038	14 (13.5)	26 (25.0)	0.035
Grade 3 or higher	9 (6.5)	15 (9.6)		6 (5.8)	10 (9.6)	
Grade 2	12 (8.7)	24 (15.4)		8 (7.7)	16 (15.4)	
Pleural effusion	6 (4.3)	22 (14.1)	0.005	4 (3.8)	14 (13.5)	0.014
Grade 3 or higher	4 (2.9)	15 (9.6)		3 (2.8)	11 (10.6)	
Grade 2	2 (1.4)	7 (4.5)		1 (1.0)	3 (2.9)	
Respiratory failure	6 (4.3)	5 (3.2)	0.603	3 (2.9)	3 (2.9)	1.000
Grade 3 or higher	3 (2.2)	4 (2.6)		2 (1.9)	3 (2.9)	
Grade 2	3 (2.2)	1 (0.6)		1 (1.0)	0	
Pulmonary embolism	1 (0.7)	1 (0.6)	0.929	1 (1.0)	1 (1.0)	1.000
Anastomotic leak	10 (7.2)	10 (6.4)	0.772	6 (5.8)	5 (4.8)	0.757
Grade 3 or higher	6 (4.3)	7 (4.5)		4 (3.9)	5 (4.8)	
Grade 2	4 (2.9)	3 (1.9)		2 (1.9)	0	
Chylothorax	2 (1.4)	1 (0.7)	0.490	2 (1.9)	1 (1.9)	0.561
Nerve paralysis	5 (3.6)	1 (0.6)	0.071	5 (4.8)	1 (1.0)	0.098
Deep vein thrombosis	1 (0.7)	2 (1.3)	0.637	1 (1.0)	1 (1.0)	1.000
Postoperative stay (days)	10 (8-12)	10 (9-12)	0.087	10 (8-12)	10 (9-12)	0.253
Chest drainage (days)	8 (6-9)	8 (7-10.5)	0.003	7.5 (6-9)	8 (7-9)	0.009
Readmitted in ICU	1 (0.7)	3 (1.9)	0.625	1 (1.0)	2 (1.9)	1.000
Unplanned reoperation	3 (2.2)	2 (1.3)	0.553	2 (1.9)	1 (1.0)	0.561
30-day mortality	1 (0.7)	0	0.469	1 (1.0)	0	0.316
90-day mortality	1 (0.7)	1 (0.6)	1.000	1 (1.0)	0	0.316
Adjuvant therapy	13 (9.4)	12 (7.7)	0.591	11 (10.6)	8 (7.7)	0.470

Categoric data are shown as number (%) and continuous data as median (interquartile range). TILO, totally minimally invasive oesophagectomy; HILO, hybrid minimally invasive oesophagectomy; ICU, intensive care unit.

**Figure 2:**
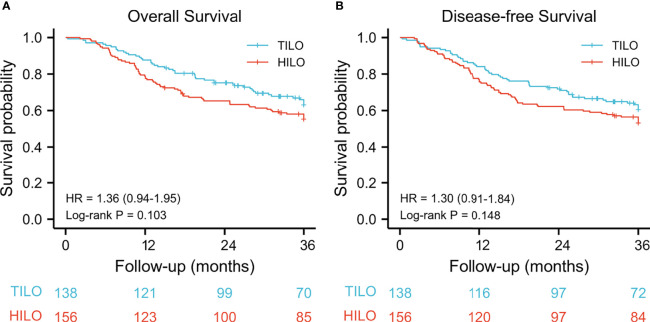
Kaplan–Meier curves for unmatched patients between totally minimally invasive oesophagectomy (TILO) group and hybrid minimally invasive oesophagectomy (HILO) group. **(A)** overall survival, **(B)** disease-free survival.

The mean follow-up time for the total population was 27.1 months (IQR 15.3–45.8) and was similar between the TILO and HILO groups (27.9 vs 26.3 months, respectively; P = 0.230). The 3-year overall survival rate was 63.1% (95% CI 55.2–72.1) for the TILO group and 55.3% (95% CI 48.0–63.8) for the HILO group with no significant difference (hazard ratio (HR) 1.36, 95% CI 0.94–1.95; P = 0.103). No statistical difference (HR 1.30, 95% CI 0.91–1.84; P = 0.148) in 3-year disease-free survival rate was found between the TILO group (60.5%, 95% CI 52.7–69.4) and the HILO group (53.0%, 95% CI 45.7–61.5) (**Figure 2**).

### Comparison of Short- and Long-Term Outcomes Between the Matched Groups

With regard to minimize confounding biases, we applied PSM analysis by matching the following covariates. A total of 104 pairs were compared, and the demographic and clinical characteristics of the matched patients are listed in **Table 1**. All standard differences (SDs) less than 0.01 indicated an adequate balance in matched population.

In this study, the estimated blood loss in the TILO group was significantly less than in the HILO group (100 vs 120 ml; P < 0.001). Conversely, the operative time in the TILO group was longer (350 vs 210 min; P < 0.001). The proximal margin length was 5.2 ± 0.2 cm in middle third, 6.3 ± 0.4 cm in lower third and 6.5 ± 0.7 cm in oesophagogastric junction tumours. No significant difference was found in margin length between TILO and HILO (6.0 ± 0.7 vs 6.0 ± 0.8 respectively; P = 0.970), which reflected the assurance of adequate surgical margin in TILO group. Notably, we found that TILO was still significantly correlated with lower morbidity rates including pneumonia (13.5 vs 25.0%; P = 0.035), pleural effusion (3.8 vs 13.5%; P = 0.014), and a shortened chest drainage (median(IQR) 7.5 (6–9) vs 8 (7–9) days; P = 0.009) compared with HILO (**Table 2**). The rates of anastomotic leak in the TILO group and HILO group were 5.8% (6/104) and 4.8% (5/104), respectively, without significant difference (P = 0.757). There was no significantly difference considering the number of harvested lymph nodes in subgroup analysis between the two groups.

**Figure 3:**
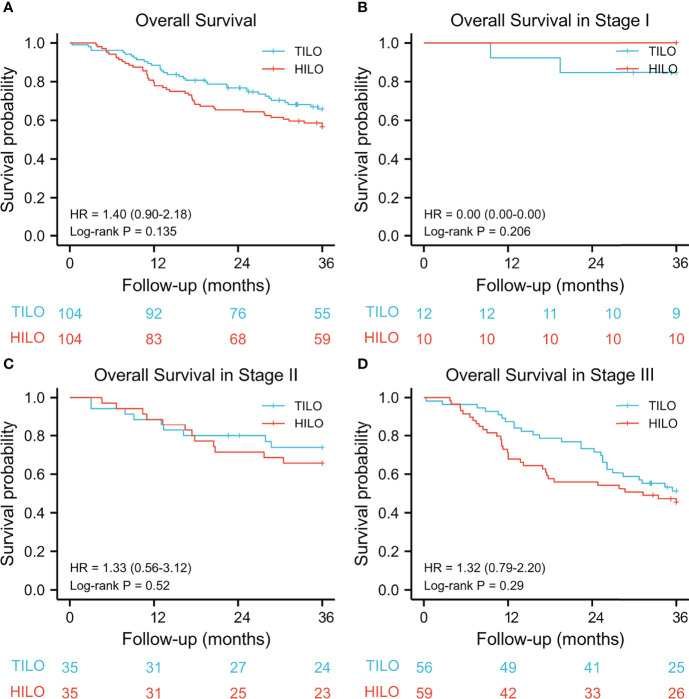
Kaplan–Meier overall survival curves stratified by clinical stage. **(A)** the entire cohort, **(B)** stage I; **(C)** stage II, **(D)** stage III.

**Figure 4:**
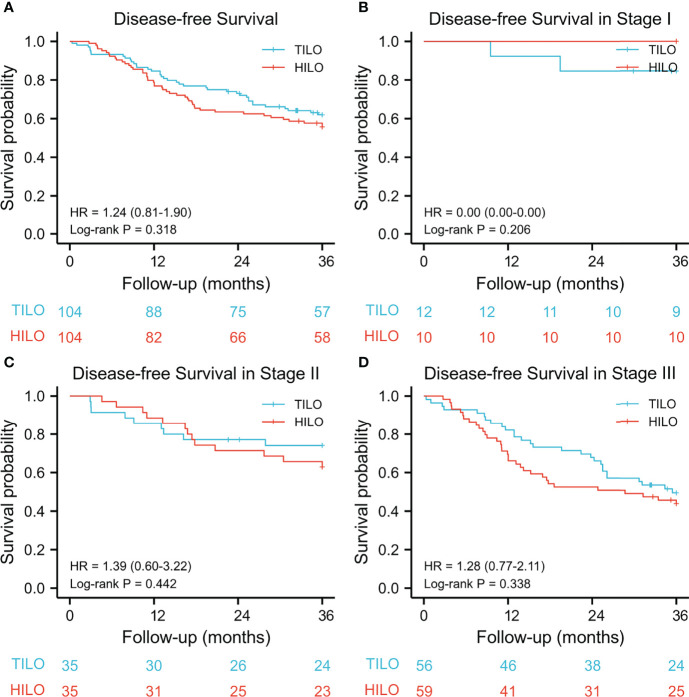
Kaplan–Meier disease-free survival curves stratified by clinical stage. **(A)** the entire cohort, **(B)** stage I; **(C)** stage II, **(D)** stage III.

The 3-year overall survival rate was 67.3% (95% CI 57.0–75.9) for the TILO group and 56.7% (95% CI 47.9–67.0) for the HILO group. The 3-year disease-free survival was 62.5% (95% CI 53.3–72.2) for the TILO group and 55.8% (95% CI 46.9–66.1) for the HILO group. In the analysis between matched groups, there were no significant differences between the TILO and HILO groups in 3-year overall survival (HR 1.40, 95% CI 0.90–2.18; P = 0.135) (**Figure 3A**) or the 3-year disease-free survival (HR 1.24, 95% CI 0.81–1.90; P = 0.318) (**Figure 4A**). The subgroup analysis showed no significant differences in overall survival rate (stage I, P = 0.206; stage II, P = 0.520; stage III, P = 0.290) (**Figures 3B–D**) and disease-free survival rate (stage I, P = 0.206; stage II, P = 0.442; stage III, P = 0.338) (**Figures 4B–D**) when patients were stratified by clinical TNM stage.

## Discussion

In this study using PSM analysis, we found that the TILO approach was associated with a lower risk of postoperative major complications including pneumonia, and pleural effusion, while the estimated blood loss and chest drainage were also reduced in this group. While the difference arrived at statistical significance in estimated blood loss, we cannot state clinical significance. The 3-year overall survival and disease-free survival rate of patients in the TILO group were similar to those in the HILO group.

Here we found that the TILO procedure was associated with a 12.5% lower incidence of postoperative pneumonia than HILO. This effect was most probably because it reduces physiological trauma and can achieve better control of postoperative pain (16, 17). A recent randomized controlled trial (16) showed that HILO resulted in a lower incidence of postoperative pulmonary complications compared with open oesophagectomy, consistent with our results. However, our study constitutes a further step toward a comprehensive understanding of how TILO can improve early postoperative outcomes in patients with oesophageal cancers. Our results are also consistent with those presented by Souche et al. (9), in which the major postoperative pulmonary complications were significantly lower in the minimally invasive oesophagectomy group compared with a hybrid surgery group. However, we had a sample size of approximately twice as many patients. Although a few studies have reported on the short-term outcomes after TILO compared with HILO, those results remain controversial (8, 9, 18, 19). Most of them reported the initial experience and aimed to prove the technique’s safety. Other studies showed no difference in postoperative pneumonia between the two procedures, which could have arisen from the limited sample size (fewer than 15 patients who underwent minimally invasive oesophagectomy) and might be explained by the surgeons’ learning curve (19). Moreover, Fumagalli et al. compared the two groups indirectly without showing the baseline data (8).

The main challenge for TILO might be the difficulties in performing intrathoracic anastomosis. However, several such techniques and experiences have been reported, including thoracoscopic hand-sewn, intrathoracic circular stapled, and robot-assisted anastomoses (20–22). In our study, no significant differences were observed between the two groups with respect to anastomotic leak. A possible reason might be that the surgeons have already passed the learning curve. TILO has been initially performed in our institution since 2011. All the surgeons were experienced in performing minimally invasive oesophagectomy including both TILO and HILO before this study conducted since 2014.

Adequate lymph node yield among node-positive patients is critical for accurate assessment of tumour staging and for improving the long-term survival in patients with oesophageal cancer (23). No statistical difference was found in the total numbers of harvested lymph nodes between the two groups in our study, but there were significantly more lymph nodes harvested in the superior mediastinum in the TILO than in the HILO groups. In our experience, thoracoscopic-assisted or robot-assisted surgery provides a high-definition field and increased spatial precision during surgery compared with conventional thoracotomy, which might be the reason for the difference.

Evidence for the long-term survival benefit of minimally invasive oesophagectomy seems limited and contradictory. Thus, Gottlieb-Vedi et al. reported long-term survival benefits after minimally invasive oesophagectomy compared with open oesophagectomy (7). However, Mariette et al. (16) concluded that there was no difference in long-term survival between patients subjected to hybrid or open oesophagectomy in a randomized controlled trial. Evidence for long-term survival comparing TILO with HILO is still deficient. In the PSM analysis, we found a trend but no significant prognostic benefit after TILO compared with HILO. Moreover, when we performed a subgroup analysis based on pathological tumour stage, no significant effects were observed in 3-year overall survival and disease-free survival rate between the two groups. The duration of follow-up time and the heterogeneity of the oesophagogastrostomy technique might weaken the significance of the survival analysis in our study.

This study has been the first to investigate whether the choice between TILO and HILO influences the long-term survival in patients with oesophageal cancers. We performed a PSM analysis in a relatively large sample size to minimize confounding bias. As a result, the study population was more homogeneous. The study by Souche et al. had a long time span of over 5 years with a smaller sample size (9). However, our study also had a relatively short time span to ensure homogeneity of surgical technique and postoperative administration. There were also some limitations in our study. First, it was retrospective and observational, with inherent flaws. Second, several studies have found evidence that anastomotic techniques might have a significant correlation with subsequent leakage (24, 25). Because of the absence of standardized intrathoracic oesophagogastrostomy approaches (26), various anastomotic techniques were used in this study, potentially increasing the bias. However, multiple factors including blood supply, anastomotic tension and diabetes, have been proposed to influence anastomotic healing. Further multicentre prospective, randomized controlled clinical trials are needed.

In conclusion, TILO resulted in improved short-term outcomes, especially less pulmonary complications, than HILO for patients with cancers of the middle or lower third of the oesophagus, with similar survival outcomes.

## Data Availability Statement

The original contributions presented in the study are included in the article/supplementary material. Further inquiries can be directed to the corresponding author.

## Ethics Statement

The studies involving human participants were reviewed and approved by the Ethics Committee of West China Hospital of Sichuan University. The patients/participants provided their written informed consent to participate in this study.

## Author Contributions

L-QC and W-PW conceptualized the study, revised the manuscript and supervised the study. Y-MG and H-LZ collected the data, drafted the manuscript and made the figures. Y-SY, YY, YH and G-WC revised the manuscript. All authors read and approved the final manuscript.

## Funding

This research was supported by Excellence Surgery Grant of Bethune Charitable Foundation (Grant No. HZB-20181119-52).

## Conflict of Interest

The authors declare that the research was conducted in the absence of any commercial or financial relationships that could be construed as a potential conflict of interest.

## Publisher’s Note

All claims expressed in this article are solely those of the authors and do not necessarily represent those of their affiliated organizations, or those of the publisher, the editors and the reviewers. Any product that may be evaluated in this article, or claim that may be made by its manufacturer, is not guaranteed or endorsed by the publisher.
